# Harmonic Shears in the Surgical Treatment of Laryngomalacia

**DOI:** 10.7759/cureus.5880

**Published:** 2019-10-10

**Authors:** Nikolay R Sapundzhiev, Lora T Nikiforova, George S Stoyanov, Ivan Valkadinov, Petya Genova, Vilian Platikanov

**Affiliations:** 1 Otolaryngology, Medical University of Varna, Varna, BGR; 2 General and Clinical Pathology, Forensic Medicine and Deontology, Medical University of Varna, Varna, BGR; 3 Anaesthesiology, Medical University of Varna, Varna, BGR

**Keywords:** laryngomalacia, supraglottoplasty, ultracision

## Abstract

Introduction

Laryngomalacia (LM) is a condition that is clinically diagnosed in the pediatric period with inspiratory stridor and is caused by a congenital or acquired collapse of laryngeal suprastructures. Endoscopic supraglottoplasty is the modern gold standard surgical treatment for severe or complicated laryngomalacia. Various cold and powered surgical devices have been used to approach the aryepiglottic folds, and their advantages and drawbacks have been widely discussed. The applicability of Ultracision Harmonic shears (Ethicon Inc., NJ, US) for the sake of supraglottoplasty has not been previously advocated in the literature and is the subject of this study.

Methods

This was a review of the medical records of pediatric patients, with moderate to severe congenital laryngomalacia, who underwent supraglottoplasty with Harmonic at a single institution, from 2013 to 2019.

Results

A total of six patients underwent bilateral aryepiglottic fold division with the use of Ultracision in the study period (4 male, 2 female; mean age 7+/-9 months, age range 1m-24m). Postoperatively, all of the children were extubated and admitted to the pediatric intensive care unit (PICU) as a precaution measure. There were no early or late complications after the intervention. The postoperative endoscopic picture was evaluated in three patients (two of which for another reason). A stable laryngeal frame with no collapse or excessive scarring was observed. None of the patients required repeat surgery.

Conclusion

Based on the ease of surgical access, performance, surgical precision, and postoperative results, the use of Harmonic scissors appears to be a safe, practical, affordable, and easily applicable alternative for supraglottoplasty Type 2.

## Introduction

Laryngomalacia (LM) is a condition that is clinically diagnosed in the pediatric period with inspiratory stridor. It is caused by a congenital or acquired collapse of laryngeal suprastructures in several distinctive patterns [1-2]. LM ranks as the most prevalent cause of stridor in infants and toddlers [3]. The exact causes of LM are still debatable. There are generally three types of LM according to the place of the most demonstrative collapse according to Olney at al. - aryepiglottic collapse (type 1), short aryepiglottic folds (type 2), and epiglottic collapse (type 3) [1]. Leading theories are based on the notion of anatomical abnormalities of the supraglottic structures [4], structural alterations of the tissues of the larynx [5-6], neurologic dysfunction [7], and inflammatory conditions [6,8]. Mild LM includes only inconsequential inspiratory stridor, moderate is complicated with feeding difficulties, and severe presents with failure to thrive and respiratory failure [9].

The standard clinical approach is observation for 12-24 months (spontaneous resolution), but in cases of severe LM or when concomitant dysphonia or dysphagia is present, precise endoscopic diagnosis and surgical treatment are required. Endoscopic supraglottoplasty is the modern gold standard surgical treatment for severe or complicated laryngomalacia that replaced tracheostomy in the 1980s. Nowadays, the instrumentation used in these types of interventions includes a cold knife, microdebriders, a coblator, or a laser [9-10]. Ultracision Harmonic shears (Ethicon Inc., NJ, US) were supposed to be useful in this condition, but no clinical evidence is reported in the literature [11].

This study reports our experiences with supraglottoplasty for Type 2 laryngomalacia, performed with a Harmonic scalpel.

## Materials and methods

Pediatric patients, with moderate to severe congenital laryngomalacia, who underwent supraglottoplasty with Harmonic at a single institution from 2013 to 2019 were retrospectively reviewed. Prior to 2013, no supraglottoplasty was performed. Indications for supraglottoplasty in this cohort included moderate to severe dyspnea with persistent or progressing stridor, sleep-disordered breathing, failure to thrive, and airway compromise on flexible or direct laryngoscopy.

Preoperative workup included a full history and physical examination with flexible laryngoscopy or direct laryngoscopy with sedation and spontaneous breathing in order to exclude other alternative or concomitant abnormalities of the upper airways that could present with inspiratory stridor. Polysomnography was unavailable. Diagnostic endoscopy with video recording was carried at least one day before the surgical intervention. The findings were discussed with the parents and informed consent for the intervention was obtained.

Surgery was performed under general anesthesia with transoral intubation. Perioperatively steroids (dexamethasone) were administered. All interventions (supraglottoplasty type 2) were performed in а uniform manner by a two-surgeon team. One surgeon manipulated a direct laryngoscope of the Macintosh type and the active instrument while the assistant held a rigid Hopkins rod lens endoscope (0° in three cases, 30° in two cases, and 0° and 70° in one case) with a direct video connection to a screen located in front of the surgical team. The powered instrument used was Harmonic ACE® shears (Ethicon Endo-Surgery, Inc.,), with a 23 mm long shaft, 5 mm shaft diameter, and a 15 mm long curved applicator. They were operated with the Harmonic Generator 300 (GEN04) with the minimum power level set at Level 3. In all six cases, bilateral sectioning of the aryepiglottic folds was performed.

Postoperatively, the children were extubated and admitted to the pediatric intensive care unit (PICU). An oral diet was started immediately after surgery. All patients were routinely given acid suppression (proton pump inhibitor, PPI) and systemic steroids. Antibiotics were neither clinically required, nor given. Early postoperative laryngoscopy was not performed in any case.

## Results

A total of six patients underwent supraglottoplasty Type 2 with the use of Ultracision in the study period. Four of them were male and two female (mean age at the time of the intervention 7+/-9 months (1m-24m). All patients were full term. The only patient with important comorbidity was a boy with achondroplasia. In four cases, the findings corresponded to LM type 2, and in two, the overlapping characteristics of laryngomalacia type 1/2 and 2/3 were observed. Secondary airway lesions were not present in any case.

**Figure 1:**
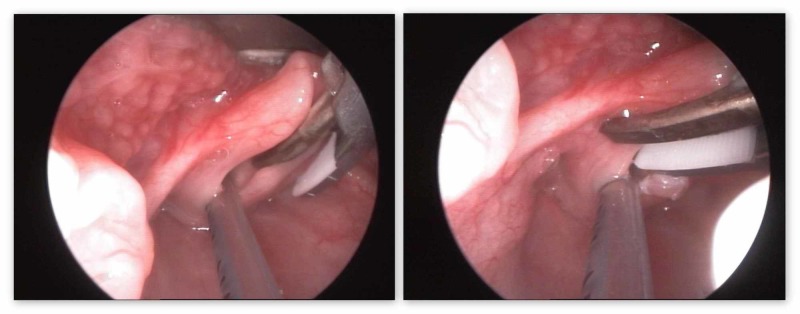
Resection of right and left aryepiglottic folds

Supraglottoplasty (aryepiglottic fold division) was performed in all cases bilaterally. The total procedure duration was a mean of 12.1 minutes with a total anesthesia time of about 40 minutes for each case. The diameter of the shaft of the Harmonic ACE® shears did not pose any difficulty for the transoral insertion of the instrument in infants and toddlers. The overall length of the instrument was judged as “appropriate” by the senior surgeon, allowing for easy and comfortable manipulation together with the direct laryngoscope in the other hand. The slightly curved asymmetrical tip of the instrument and the rotatable shaft helped to work along the axis of the airway in the sagittal plane. These features, however, could not be used to help grasp structures on the left and right sides of the larynx. Thus, the right aryepiglottic fold was transected with the metal blade on the inside of the larynx and the silicon-coated blade on the outside of the larynx while on the left side, the metal blade was on the outside and the silicone one on the inside of the larynx (Figure 1). This somehow interferes with the symmetrical visualization and transection on both sides. No endotracheal tube injury occurred. The size of the blades was found appropriate for the fine structures of the pediatric larynx. No signs of bleeding were apparent after a single activation of the powered instrument. All patients were successfully extubated in the operating room; non-problematic spontaneous respiration with immediate improvement/resolution of the stridor was noted. No intraoperative or direct postoperative complications were observed.

All patients were monitored overnight in the PICU, transferred to a standard pediatric or ear, nose, throat (ENT) ward for further 24-hours observation, and discharged the following day. The patients began PO intake a few hours after surgery without any signs of dysphagia or aspiration. There were no complications in the immediate postoperative period. No patients required reintubation, non-invasive ventilation, or other respiratory support, apart from an oxygen mask. This oxygen supplementation was not indicated by abnormal values of the saturation but is rather routinely applied to all infants and toddlers in the postoperative setting in the PICU.

Control endoscopy was not adopted as a standard follow-up strategy due to ethical concerns. It would have been very difficult to persuade the parents for another intervention (even flexible endoscopy) in the absence of any significant residual symptoms. Still, in one of the cases, the postoperative status was evaluated endoscopically two months postoperatively. The reason for this was minimal positional inspiratory stridor. Control laryngoscopy with sedation and spontaneous breathing did not show collapse or any instability of the supraglottic structures. The aryepiglottic folds seemed shorter again. No further intervention was deemed necessary. The child was discharged and weeks later, the parents reported that the positional inspiratory sound had disappeared. Thus, none of the patients required repeat surgery, as the residual symptoms were insignificant if any. Two other patients, however, necessitated general anesthesia for adenoid surgery two years and five years after supraglottoplasty, respectively. Along with the main intervention, the late outcome from the supraglottoplasty was evaluated via rigid laryngoscopy. In one of the patients, the scars from the aryepiglottic folds surgery were visible and redundant arytenoid mucosa was present. In both cases, however, the laryngeal frame was stable enough, the intubation was uneventful, and no supraglottic collapse was detected.

## Discussion

In this case series, pediatric patients with moderate to severe congenital laryngomalacia were treated surgically using a novel device - Harmonic shears. Endoscopic surgery is an established approach for children with laryngomalacia and moderate or severe respiratory symptoms or failure to thrive [2]. The variety of interventions (apart of tracheostomy) in the literature may practically be summarized into three major groups of supraglottoplasty: Type 1 - debulking of arytenoids; Type 2 - aryepiglottic folds division; and Type 3 - epiglottis surgery with few distinctive subtypes. This reflects the well-detailed classification of LM itself [1,12].

Clinical outcome

The nature of the Harmonic scalpel allowed for fast and effective surgery with no bleeding. There were no complications during surgery and anesthesia. No signs of local edema with impaired respiration could be clinically perceived. Patient recovery and restoration of normal laryngeal function were quick and stable. All patients had sufficient improvement with only minor residual clinical symptoms in one case. There were no significant complications. None of the patients in our series required repeat surgery, as all initial interventions were bilateral. Initial bilateral supraglottoplasty is associated with a four-fold lower risk for a second operative procedure [13-14]. The only case of a boy with any important associated comorbidity (achondroplasia with potential disproportions of the upper airways) also recovered uneventfully and presented no further laryngomalacia-related clinical signs.

Endoscopy for evaluation

No early or routine postoperative endoscopic evaluation was performed, as none of the patients exhibited any alarming clinical signs, such as stridor, respiratory distress, and/or obstructive airway symptoms, suggestive of residual laryngomalacia. This prevented us from monitoring the wound healing process and estimating the type and degree of mature postoperative changes. The clinical evaluation of respiration and feeding was the only criteria for discharging children. Other studies also report that postoperative flexible fiberoptic laryngoscopy is seldom performed and quite preferred [11]. The absence of postoperative endoscopic evaluation due to ethical concerns may be regarded as a weakness of our study. The only case that was subjected to repeat laryngoscopy two months after the intervention showed the stability of the aryepiglottic tissues. No scarring of the mucosa was noticeable, but the aryepiglottic folds seemed shorter than expected without a marked V-shaped wedge.

Tool wound healing

This is the first case series describing the outcomes of supraglottoplasty using Harmonic shears. In a study on the preferences for supraglottoplasty techniques in the US, the use of this tool was included in the options list in the survey as a viable option. The majority of respondents (83%) use only cold steel, of which 10% use a microdebrider as a powered cold steel instrument. Only 3% use a coblator as a low-temperature device achieving hemostasis while simultaneously ablating tissue. Fourteen percent use a laser. Yet, none of the respondents used a Harmonic powered instrument (11). We did not find any other publications reporting the results of the use of this type of powered instrument in supraglottoplasty.

With the Harmonic instruments, a longitudinal vibration at 55.5 kHz at the blade causes denaturation of the collagen molecules, forming a coagulum. So the vibration causes both the cutting and sealing of small vessels by the coagulated blood and tissue proteins. The Harmonic scalpel has proven to be a viable alternative to classical and other modern surgical tools, as well as in surgery carried out in the head and neck region (14-17). The Harmonic shears seem to be excellent for supraglottoplasty Type 2 at achieving hemostasis while simultaneously ablating tissue, which seems to be an advantage over the cold steel technique.

Tool size

Supraglottoplasty usually is performed by suspension microlaryngoscopy with intubation [6,10,15-23] or without intubation [22-28]. The setting provides optimal visualization but is resource and time-consuming, especially if using a microscope-mounted CО2 laser system with the appropriate laser-resistant tracheal tubes. Handheld direct laryngoscopes of the Miller type represent an option for visualization of the larynx in this type of surgery [23]. It can be used with rigid 0o rigid telescopes for pediatric laryngeal surgery. The optic may be attached to the laryngoscope blade. This partially reduces the need for a second surgeon but limits the use of the telescope partially as a retractor or repositioning it to give another aspect at the operating field, without displacing the Miller blade. With this approach, the surgical time for supraglottoplasty (not specified type) is reduced to 2-3 minutes. We used the direct laryngoscope of the Macintosh type in association with both the 0o and 30o rigid scopes. The scope was held by a second surgeon, which allowed for a very smooth change of the aspect at the surgical field. This, together with the slightly curved tip of the Harmonic shears, allowed us to work to some degree off the axis of the airway with less occipital deflection of the child’s head. The choice for laterality in unilateral supraglottoplasty may be based on the subjective preferences of the surgeon, right- or left-handedness or the construction of the applicator. Cold steel instruments usually have paired left and right curved forceps and scissors. The CO2 laser may be used in a perfectly symmetrical way and has the advantage of being incorporeal and leaving more space in the operative field. Bipolar radiofrequency ablation devices of the coblator type have a symmetrical shape, allowing for an equal approach to the left or right aryepiglottic fold [10]. The Harmonic shears have a rotatable shaft, the curved blades may help to resect the aryepiglottic fold perpendicularly but do not allow for perfectly symmetrical grasping of the structures on the left and on the right of the larynx. The right aryepiglottic fold is grasped with the metal blade on the inside of the larynx and the silicon-coated blade on the outside of the larynx while the left fold is grasped with the metal blade was on the outside and the silicone one on the inside. In this aspect, the use of an angulated endoscope that is freely movable by the assistant helped us achieve optimal visualization and compensate for the asymmetry of the powered instrument.

Supraglottoplasty may be performed with instrument sets smaller in size than the conventional sets for microlaryngeal surgery. Tunkel prefers using conventional sinus instruments as compared to almost twice as long standard microlaryngeal scissors both with an operating microscope or a rigid telescope [19]. Even smaller instruments have been used for Type 1 supraglottoplasty - otologic micro-cup forceps and Bellucci scissors [23]. Our procedure protocol proved to be easy to perform in patients of this age and size. The size and shape of the Harmonic scalpel shaft as a whole did not pose any difficulty, with the transoral approach in an intubated child, a direct Macintosh type laryngoscope in place for positioning and a 4 mm 175 mm rigid Hopkins rod lens endoscope coupled to a camera for visualization. The applicator of the Harmonic shears proved to be appropriate in shape and size for the procedure, regarding the size of the laryngeal complex and the volume of tissue resection needed in patients of this age. Five of six children in our series were of normal weight and stature. We used the Harmonic shears with ease also in the case with achondroplasia, where the boy had shorter limbs but apparently normal proportions of the facial skeleton and neck. However, we assume the size of the applicator might pose some difficulties in premature and low birth weight babies with smaller laryngeal complexes.

Fire

During laryngeal and hypopharyngeal surgery by powered instruments, ignition of the intubation tube is possible, as has been reported with different types of lasers and monopolar electrocautery [29]. Bipolar radiofrequency ablation devices are believed to be safer in terms of airway fire as compared to the CO2 laser [10]. In 15 procedures with coblation supraglottoplasty with the endotracheal tube in place, tube damage did not occur [10]. Still, this particular issue has never been addressed in an experimental model. The Ultracision Harmonic shears appear to be aggressive to polyvinyl chloride (PVC) endotracheal tubes. They may be perforated or the inflation cuff of the tube may be damaged by the ultrasound device, leading to leaks. Ignition of a standard PVC endotracheal tube by Ultracision Harmonic shears appears to be impossible even with high-flow oxygen [30]. Particularly in supraglottoplasty Type 2, the use of Ultracision shears seems to provide more safety for inadvertent tubes damage by airway fire.

The limitations of this study include those associated with retrospective case series. Swallowing outcomes were not measured objectively, wound healing was not monitored, and the postoperative evaluation was based only on a clinical basis, with occasional endoscopies performed at later stages. This is also a very small series, which limits the comparison of outcomes to other studies of supraglottoplasty. Only patients with Type 2 supraglottoplasty (aryepiglottic folds division) were included. Nevertheless, there is evidence that Type 2 supraglottoplasty may be effective in improving LM symptoms irrespective of its type in 90% of the cases [26].

## Conclusions

The application of the Harmonic scalpel in supraglottoplasty appears promising, with the potential of optimized hemostasis, which is one advantage over the cold steel technique. Fire safety is another important advantage, particularly over the laser. The Harmonic shears may be used for bilateral sectioning of the aryepiglottic folds, though the asymmetrical construction of the instrument makes it very different working on the left and right sides. The proposed surgical protocol for supraglottoplasty with Harmonic scissors does not deviate from the already classically established ones. Based on the ease of surgical access, performance, surgical precision, and postoperative results, the use of the Harmonic scissors appears to be a safe, practical, affordable, and easily applicable alternative for supraglottoplasty Type 2.
